# The Relationship between Depression and Asthma: A Meta-Analysis of Prospective Studies

**DOI:** 10.1371/journal.pone.0132424

**Published:** 2015-07-21

**Authors:** Yong-hua Gao, Hua-si Zhao, Fu-rui Zhang, Yang Gao, Pamela Shen, Rong-chang Chen, Guo-jun Zhang

**Affiliations:** 1 Department of Respiratory and Critical Care Medicine, The First Affiliated Hospital of Zhengzhou University, Zhengzhou, Henan, China; 2 State Key Laboratory of Respiratory Disease, National Clinical Research Center for Respiratory Disease, Guangzhou Institute of Respiratory Diseases, The First Affiliated Hospital of Guangzhou Medical University, Guangzhou, Guangdong, China; 3 Medical Sciences Graduate Program, McMaster University, Hamilton, Ontario, Canada; National Cancer Center, JAPAN

## Abstract

**Background:**

Previous studies have suggested that asthmatic patients often have comorbid depression; however, temporal associations remain unclear.

**Objectives:**

To determine whether depression predicts asthma and, conversely, whether asthma predicts depression.

**Methods:**

A literature search was conducted without language restrictions using Pubmed, Embase, Cochrane and PsycINFO for studies published before January, 2015. Papers referenced by the obtained articles were also reviewed. Only comparative prospective studies with reported risk estimates of the association between depression and asthma were included. In order to investigate whether one of these conditions was predictive of the other, studies were excluded if enrolled participants had pre-existing depression or asthma. A random-effects model was used to calculate the pooled risk estimates for two outcomes: depression predicting asthma and asthma predicting depression.

**Results:**

Seven citations, derived from 8 cohort studies, met our inclusion criteria. Of these, six studies reported that depression predicted incident adult-onset asthma, including 83684 participants and 2334 incident cases followed for 8 to 20 years. Conversely, two studies reported that asthma predicted incident depression. These studies involved 25566 participants and 2655 incident cases followed for 10 and 20 years, respectively. The pooled adjusted relative risks (RRs) of acquiring asthma associated with baseline depression was 1.43 (95% CI, 1.28–1.61) (P<0.001). The adjusted RRs for acquiring depression associated with baseline asthma was 1.23 (95% CI, 0.72–2.10) (P = 0.45).

**Conclusions:**

Depression was associated with a 43% increased risk of developing adult-onset asthma. However, asthma did not increase the risk of depression based on limited studies. Further prospective studies ascertaining the true association between asthma and subsequent risk of depression are warranted.

## Introduction

Depression and asthma are two highly prevalent chronic diseases in the United States and worldwide, imposing unacceptable social and economic burdens on the public healthcare system [1,2]. Approximately 16% of adults in the United States are diagnosed with major depression disorder, and 5.8% of men and 9.5% of women will likely experience an episode of depression within a 12-month period [3]. Equally detrimental, asthma affects 39.5 million Americans, 29.0 million of which are adults, and 300 million individuals worldwide [4], with increasing prevalence in many countries [5]. Because both depression and asthma impose substantial public health burdens, the association between these two conditions has attracted attention over the past several decades.

A number of prospective studies have assessed the temporal association between depression and asthma; however, the results were inconclusive. A previous meta-analysis of prospective studies [6] reported a bidirectional relationship between psychosocial factors and atopic disorders. However, this meta-analysis only included studies published before 2007, and was lacking in studies which specifically address the relationship between depression and asthma (there were only two investigating depression predicting asthma and none examined asthma predicting depression). Since then, many more well-designed prospective studies have been published [7–9], allowing for a more detailed analysis of the temporal relationship between these two illness.

Therefore, the aim of this study was to systematically examine whether depression predicts asthma and, conversely, whether asthma predicts depression by conducting a meta-analysis of prospective studies.

## Materials and Methods

### Literature search

Two authors (Y.H.G. and H.S.Z.) searched the Pubmed, Embase, Cochrane and PsycINFO databases for relevant articles published before January 2015 using the search terms “depression,” “depressive symptoms,” and “asthma”, "wheeze" combined with “cohort studies,” “follow-up studies,” "longitudinal studies" and “prospective studies” without language restrictions. In addition, we reviewed references of obtained articles and previous meta-analyses for additional publications.

### Study Selection

Studies were eligible for analysis if they met all of the following criteria: (1) the studies were of prospective design; (2) the exposure was depression or depression symptoms (for depression predicting asthma), or asthma (for asthma predicting depression); (3) the end point was incident asthma (for depression predicting asthma), or onset of depression (for asthma predicting depression); (4) the studies excluded prevalent cases of either depression (for asthma predicting depression onset) or asthma (for depression predicting asthma onset); (5) there was sufficient data generated to make a relative risk estimate with 95% confidence intervals (CIs). When multiple publications from the same study population were available, we included the most recent publication.

Abstracts published in scientific conferences or website materials were excluded, because these studies have not been peer-reviewed and their inclusion may bias the results of a meta-analysis.

### Data extraction

We extracted data from selected articles, with particular regards to: the last name of the first author, publication year, country of region, study population, follow-up time, number of cases and size of the cohort, measurements of depression and asthma, the most fully adjusted risk estimate and corresponding 95% CI, and statistical adjustment for the main confounding or mediating factors.

We assessed the quality of each included study using the Newcastle-Ottawa Quality Assessment Scale for cohort studies [10] to determine the quality of selection, comparability, exposure, and outcome of study participants, giving a maximum of 9 points. Two authors (Y.H.G. and H.S.Z.) independently extracted the data and evaluated the study quality, with disagreements resolved through mutual discussion.

### Statistical Analysis

The RRs were used as the common measure of association between depression and asthma across studies. The hazard ratios (HRs) and odds ratios (ORs) were directly considered equivalent to RRs. Two separate analyses were conducted: depression predicting asthma, and asthma predicting depression. If a study only presented stratified risk estimates (i.e. smoking status) [11], we combined the estimates using a random-effects model and then the pooled estimate was used for the meta-analyses. For studies presenting with graded relationships (i.e. low, medium, high depression symptoms) [11,12], we only used the estimate for the highest category.

Heterogeneity across the studies were tested by using the *I* ^*2*^ statistic [13], which is a quantitative measure of inconsistency across studies, with suggested thresholds for low (25%-50%),moderate (50%-70%) and high (>75%) heterogeneity, respectively. A random-effects model, which considered both within-study and between-study variation, was used to obtain the combined risk estimates regardless of heterogeneity. Given that the studies differed in sample characteristics (i.e. sex, age and race), depression measure, asthma diagnosis, degree of adjustment, follow-up periods, we further conducted sensitivity analyses to explore possible explanations and to examine the robustness of the pooled risk estimates based on various exclusion criteria. We also investigated the impact of a single study on the overall pooled estimate by omitting one single study at a time and recalculating the pooled effect estimate of other remaining studies.

Potential publication bias was assessed by visual inspection of the funnel plot in which the log RRs were plotted against their standard error. The sensitivity analyses and publication bias were performed only for depression predicting asthma but not asthma predicting depression due to the small numbers of studies available. Begg's and Egger's test were used to estimate the severity of publication bias, with a P value <0.05 considered statistically significant. Statistical analysis was performed using Stata 12.0 (Stata Corp, College Station, Texas, USA) and Cochrane Collaboration Review Manager 5.1.2 (Cochrane Collaboration, Oxford, UK) software.

## Results

### Literature search

**Fig 1:**
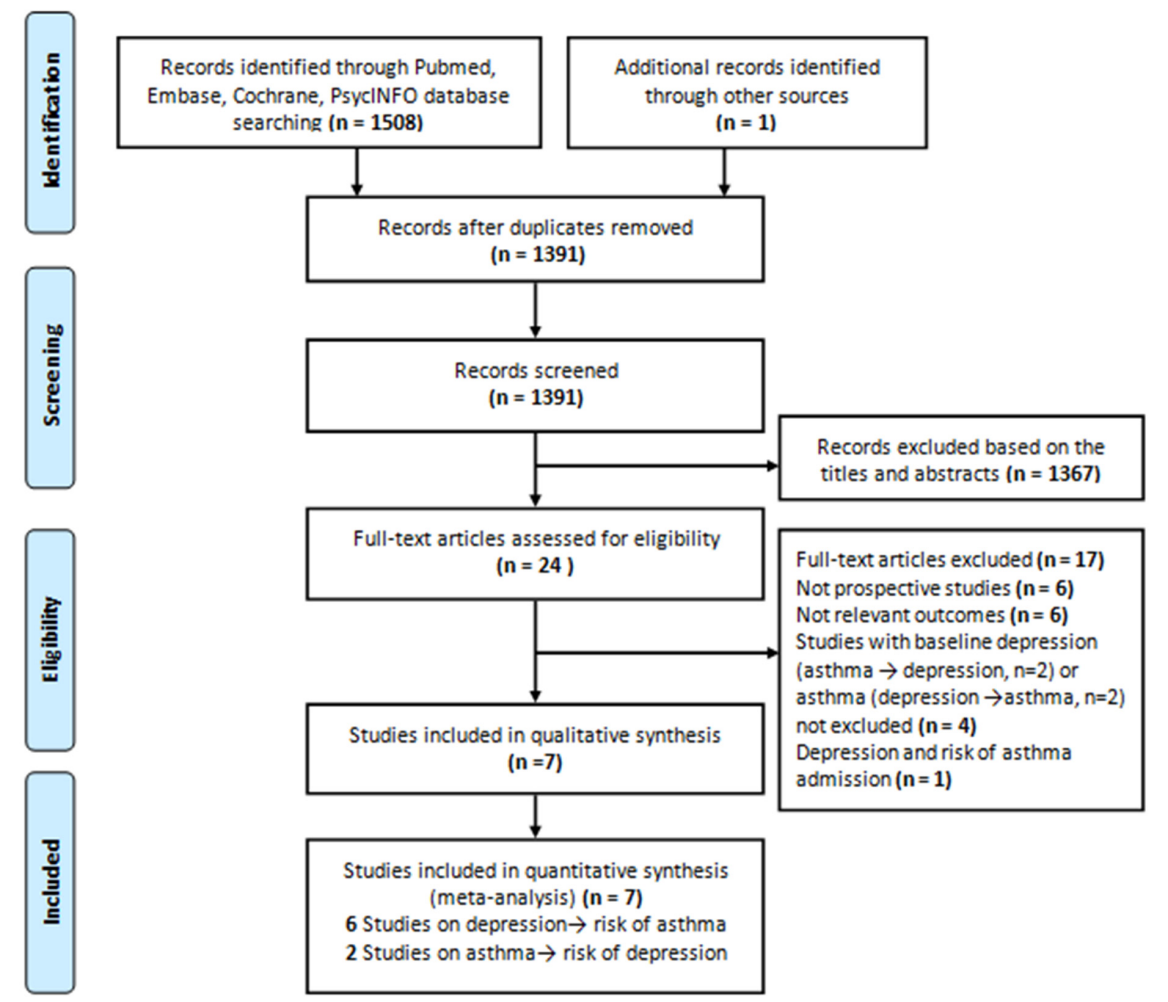
Flow chart showing the procedure for identifying the studies that were included in the meta-analysis.

A total of 1390 citations were retrieved from electronic databases. After initial screening of titles and abstracts utilizing the aforementioned criteria, 23 articles were identified for full-text review. Of these, 17 were further excluded, leaving 6 eligible articles. Hand searching of references listed within these articles identified one additional article. Seven articles were included in the final meta-analysis [7–9,11,12,14,15]. Among the included articles, five studies specifically reported results on depression predicting asthma [8,9,11,12,14].One study examined asthma predicting depression [15], and another study looked at both depression predicting asthma and asthma predicting depression [7]. A flow chart of the literature search and detailed reasons for exclusion of the other 17 studies are shown in Fig 1 and S1 Table, respectively.

### Depression predicting asthma risk

Table 1 presented the characteristics of six studies examining whether depression predicts onset of asthma. These studies were published between 1999 and 2014. Of these, three studies were conducted in the United States [7,8,11], two in European countries [9,12], and one in Canada [14]. Follow up duration ranged from 8 to 20 years across studies, with a median of 11.5 years. Five studies were conducted in both sexes [7,9,11,12,14], and one only in women [8]. The sample sizes ranged from 3,614 to 31,848, resulting in a total of 83684 participants and 2334 incident cases across studies. In defining depression, five studies used a self-reported symptoms scale [7–9,11,12], and one other used a structured clinical diagnostic interview [14]. Asthma was identified by self-report in all selected studies [7–9,11,12,14]. Participants with asthma at baseline were excluded in all six studies. To control for confounding factors, all of the included studies were adjusted for age, sex, and half of them were also adjusted for smoking and bodymass index (Table 2). The quality scores varied from 7 to 8 points according to the Newcastle-Ottawa Quality Assessment Scale, with a median of 8 points (Table 3).

**Table 1:** Characteristics of included prospective studies reviewed.

Sources	Study participants	Duration, y	Depression assessment	Asthma Ascertainment	No. of cases
**Studies for depression predicting incident asthma**					
Coogan PF et al, 2014 [8]	31,848 African American women aged 21–69 y in the United States	12	20-Item CES-D≥16	Self-report physician-diagnosed asthma (asthma was defined as a firstdiagnosis of asthma with concurrent use of asthma medication	771
Brunner WM et al, 2014 [7]	3614 men and women aged 23–35 y in the United States	Mean 20	20-Item CES-D≥16	Self-reported provider-diagnosed asthma (asthma was defined by a new report of asthma medication use and/or self-reported provider diagnosis of asthma)	429
Brumpton BM et al, 2013 [9]	23599 men and women aged 19–55 y in the Norway	11	14-Item HADS-D≥8	Self-report	890
Loerbroks A et al, 2010 [12]	5114 women and men aged 40–65 years in the Germany	Median 8.5	Paranoid Depressiveness Scale	self-report	63
Patten SB et al, 2008 [14]	14278 women and men aged over 12 y in the Canada	8	Composite International Diagnostic Interview Short Form (CIDI-SF)	Self-report	NA
Jonas BS et al, 1999 [11]	5231 women and men aged 25–74 y in the United States	Mean 9.4	GWB-D, score 0–12	Self-report	181
**Studies for asthma predicting risk of depression**					
Brunner WM et al, 2014 [7]	3016 men and women aged 23–35 y in the United States	20	20-Item CES-D≥16	Self-reported provider-diagnosed asthma (asthma was defined by a new report of asthma medication use and/or self-reported provider diagnosis of asthma)	903
Walters P 2011 [15]	22550 men and women aged over 16 y in UK	10	medical diagnosis (defined by Read/OXMIS codes)	medical diagnosis	1752

Abbreviations: CES-D = the Center for Epidemiological Studies-Depression Scale;CIDI-SF = Composite International Diagnostic Interview Short Form; GWB-D = General Well-Being Schedule-depression scale; HADS-D = the Hospital Anxiety and Depression Scale-Depression scale;NA = Not available; y = year.

**Table 2:** Adjustment for potential confounding factors.

Author, year	Adjustment
**Studies for depression predicting incident asthma**	
Coogan PF et al, 2014 [8]	Age, calendar time, BMI, femalehormone use, presence of sleep apnea, income, pack-years of smoking
Brunner WM et al, 2014 [7]	Age, sex, race, education, physical activity, study center, smoking status, BMI
Brumpton BM et al, 2013 [9]	Age, sex, smoking, physical activity, family history of asthma, education, social benefit and economic difficulties, BMI
Loerbroks A et al, 2010 [12]	Age, sex, education, smoking status, alcohol consumption, BMI, physical exercise, family history of asthma
Patten SB et al, 2008 [14]	Age, sex, health care use
Jonas BS et al, 1999 [11]	Age, sex, race, education, poverty index, urban versus rural residence, respiratory symptoms, and predicted to observed FEV_1_ ratio
**Studies for asthma predicting risk of depression**	
Brunner WM et al, 2014 [7]	Age, sex, race, education, physical activity, study center, smoking status, BMI
Walters P et al, 2011 [15]	Diabetes, cardiovascular disease, cerebrovascular disease, smoking status, age and sex

Abbreviations: BMI = Body-mass index, FEV_1_ = Forced expiratory volume in one second.

**Table 3:** Assessment of study quality^1^ included in the meta-analysis.

Source	Selection				Comparability^a^		Outcome		Total scores
	1	2	3	4	5A	5B	6	7^b^	8^c^	
**Depression predicting asthma**										
Coogan PF et al, 2014 [8]	★	★	★	★	★	★	-	★	★	8
Brunner WM et al, 2014 [7]	★	★	★	★	★	★	-	★	★	8
Brumpton BM et al, 2013 [9]	★	★	★	★	★	★	-	★	★	8
Loerbroks A et al, 2010 [12]	★	★	★	★	★	★	-	★	★	8
Patten SB et al, 2008 [14]	★	★	★	★	★	-	-	★	★	7
Jonas BS et al, 1999 [11]	★	★	★	★	★	-	-	★	★	7
**Asthma predicting depression**										
Brunner WM et al, 2014 [7]	★	★	-	★	★	★	★	★	★	8
Walters P et al, 2011 [15]	★	★	★	★	★	★	★	★	★	9

^1^ Representativeness of the exposed cohort; 2 selection of the non-exposed cohort; 3 ascertainment of exposure; 4 demonstration that outcome interest was not present at start of study; 5 comparability of cohorts on the basis of the design or analysis; 6 assessment of outcome; 7 was follow-up long enough for outcomes to occur; 8 adequacy of follow-up cohorts

^a^ Studies that controlled for age received one score, whereas studies that controlled for other important confounders received an additional score

^b^ Study with follow-up time >5 years was assigned one score

^c^ Study with follow-up rate >70% was assigned one score

**Fig 2:**
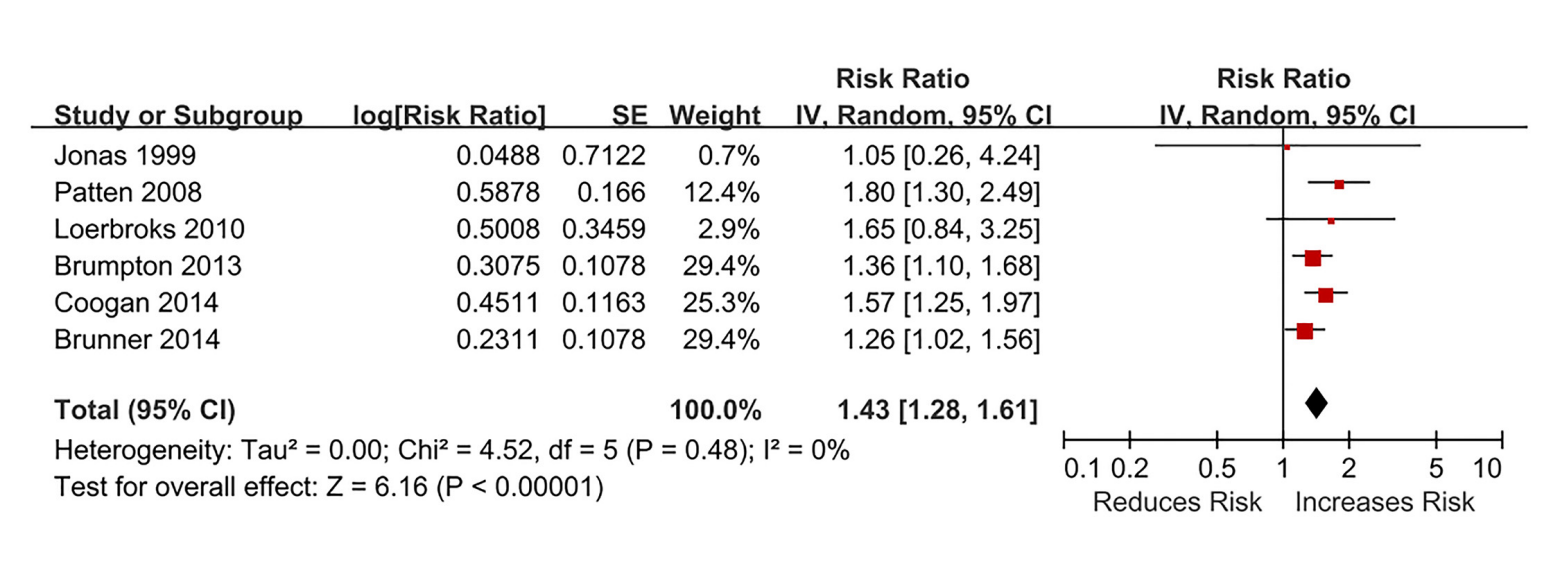
Association between depression at baseline and the subsequent risk of adult-onset asthma.

Fig 2 presents adjusted RRs with 95% CIs for all six studies assessing the association between depression and risk of incident asthma. All individual studies reported positive associations (i.e. RR>1.00), with four of them being statistically significant. The pooled RR of 1.43 (95% CI, 1.28–1.61) (P < 0.001) shows that depression was associated with increased risk of developing asthma, with no heterogeneity detected (I^2^ = 0%, P = 0.48).

For the sensitivity analyses (Table 4), the final results did not materially change for various exclusion criteria. The exclusion of any single study also did not alter the overall combined RR, with a range of 1.39 (95% CI, 1.22–1.59) to 1.51 (95% CI, 1.32–1.73).

**Table 4:** Sensitivity Analyses Based on Various Exclusion Criteria for depression predicting incident adult-onset asthma.

Outcome	No. Studies	No. Participants	No. Cases	RR (95% CI)	P Value	I^2^, %	P value for heterogeneity
All included studies	6	83684	2334	1.43 (1.28–1.61)	<0.001	0	0.48
Large-scale studies (number >5000)	5	80070	1905	1.49 (1.30–1.71)	<0.001	0	0.77
Long-term follow-up durations (>10 y)	3	59061	2090	1.38 (1.22–1.57)	<0.001	0	0.38
Studies adjusted for smoking status and BMI	4	64175	2153	1.39 (1.23–1.57)	<0.001	0	0.53
Studies adjusted for family history of asthma	2	28713	953	1.38 (1.13–1.69)	<0.001	0	0.59

### Asthma predicting depression risk

Two cohort studies investigating whether baseline asthma predicted future risk of incident depression were included [7,15], with study characteristics and adjusted confounding factors shown in Table 1 and Table 2, respectively. The first study [7] was a prospective cohort study in the United States with a follow up period of 20 years and 3016 participants aged 23 to 35 years. Asthma was diagnosed by self-report, and depression was defined by a self-reported symptom scale. The quality score was 8 points according to the Newcastle-Ottawa Quality Assessment Scale (Table 3). The second study [15] was a historical cohort study in the United Kingdom enrolling 22550 participants aged 16 years or older. The duration of follow-up was 10 years. Asthma at baseline was defined by a clinical diagnosis, and depression was defined by a medical diagnosis from Read/OXMIS codes. The quality score was 9 points (Table 3). The two studies were conducted in both sexes, and participants with depression at baseline were excluded.

**Fig 3:**
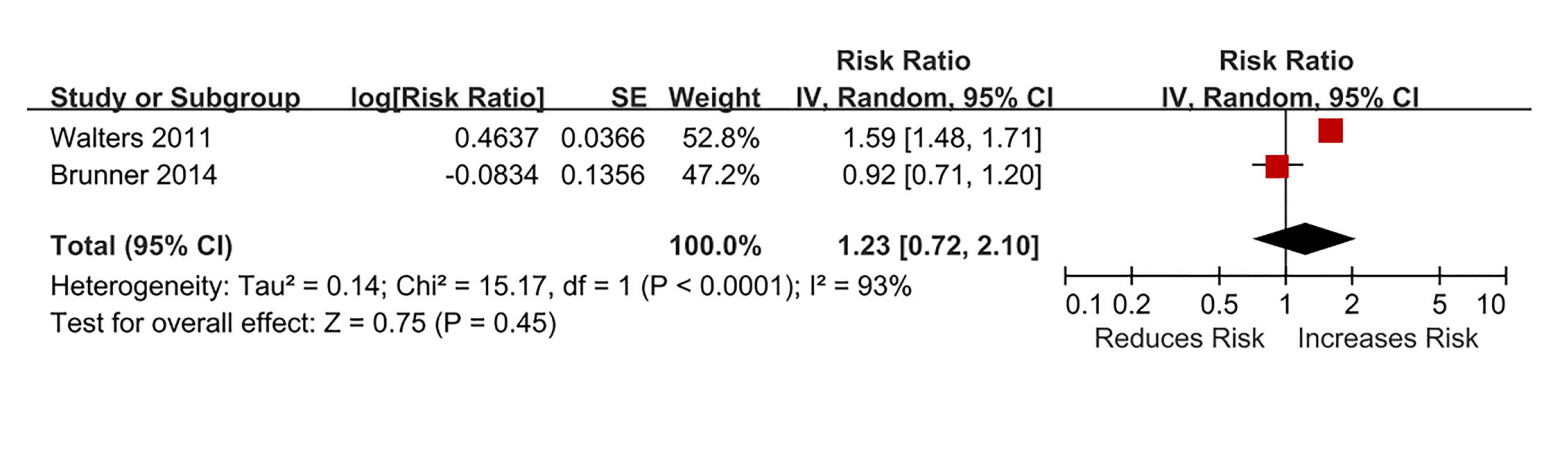
Association between asthma at baseline and the subsequent risk of depression.

The pooled adjusted RR was 1.23 (95% CI, 0.72–2.10) (P = 0.45) (Fig 3) with high detected heterogeneity (I^2^ = 93%, P < 0.0001). The stratified and sensitivity analyses were not performed because only two studies were available for analysis.

### Publication bias

**Fig 4:**
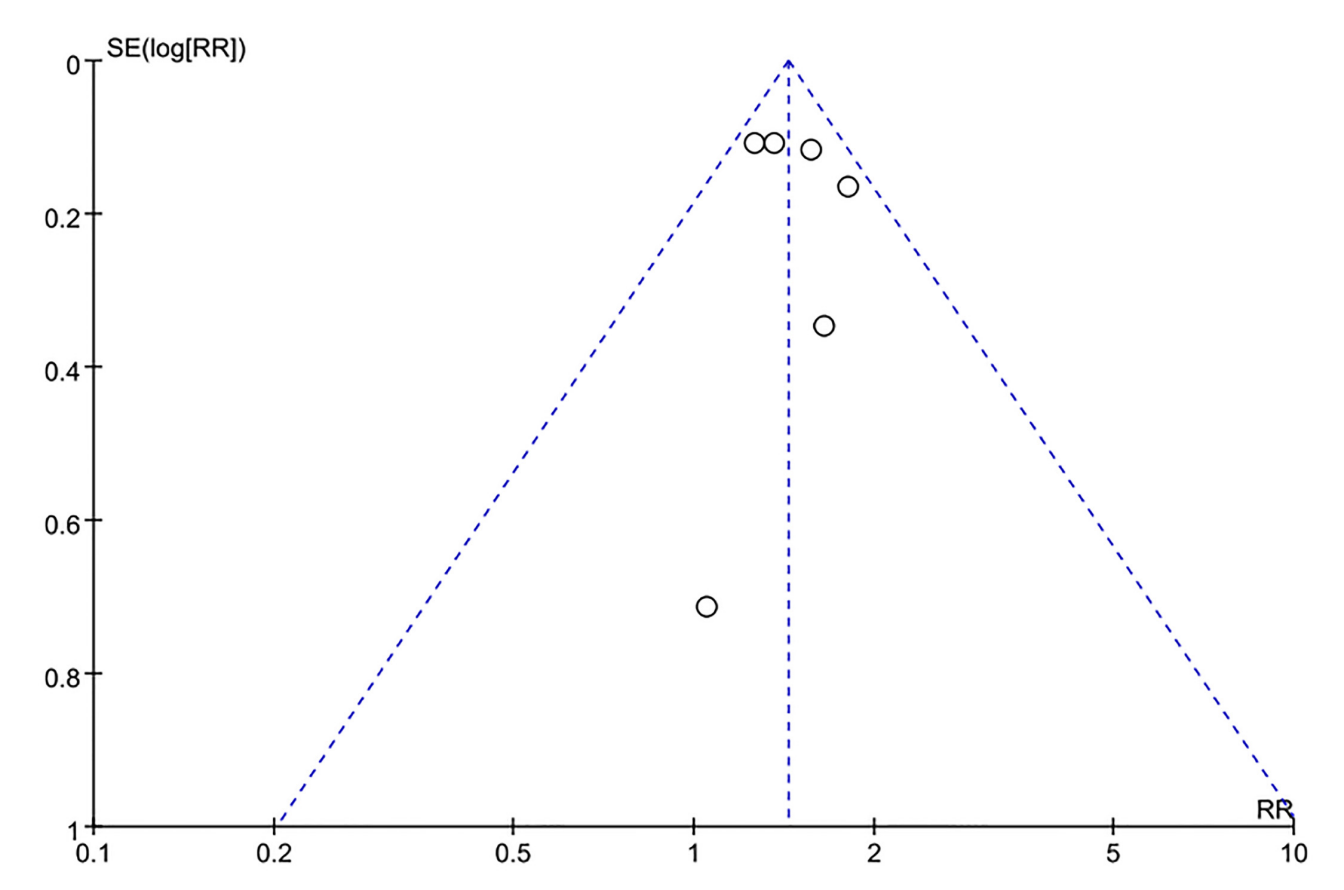
Funnel plots for assessment of publication bias among studies included in the meta-analysis of the association between depression at baseline and the subsequent risk of adult-onset asthma.

Visual inspection of the funnel plot indicated some asymmetry for depression at baseline predicting adult-onset asthma (Fig 4). However, Begg's and Egger's test did not show significant evidence of publication bias for studies of depression predicting asthma (Begg's test, P = 0.21; Egger's test, P = 0.15). Publication bias for asthma predicting incident depression was not assessed due to the limited number of studies.

## Discussion

To the best of our knowledge, this is the first meta-analysis that examines the temporal association between depression and asthma using data from prospective studies. The results indicate a strong and robust association between depression and incidence of adult-onset asthma after adjustment for potential confounding factors. On the other hand, no association was found between asthma and subsequent risk of depression, but this may have resulted from too few studies available.

Our current meta-analysis, based on stringent inclusion criteria, provides strong evidence that depression is associated with increased risk of asthma without heterogeneity among studies (I^2^ = 0, P = 0.48).This is in agreement with a recent meta-analysis of four cross-sectional studies (OR, 3.17; 95% CI, 2.82–3.56) [16]. In an attempt to produce more precise pooled estimates, we only included prospective studies that clearly stated the enrollment of patients without comorbid asthma at baseline. In addition, exclusion of any single study and sensitivity analyses based on various exclusion criteria did not materially alter the final results, which increased the robustness of our findings. All of this increased the strength and reliability of our final conclusion. Therefore, the association between depressive symptoms and incident asthma is clear. Further studies elucidating whether prevention or effective treatment of depression may have implications for asthma prevention in addition to psychological functioning and well-being are warranted.

In contrast to depression predicting incident asthma, this meta-analysis examining the effect of asthma on incident depression showed a trend toward increased RRs but did not reach significant difference statistically with high heterogeneity. This may be mainly due to the limited number of high-quality studies included. Although we identified 2 additional prospective studies that could be pooled. they did not exclude participants who reported depressive symptoms at baseline [17,18]. Therefore, we chose to err on the side of having imprecise but unbiased estimates rather than having precise but potentially misleading estimates. Of the two additional studies, one reported that asthma in early adolescence was associated with an elevated risk of developing major depression (HR, 1.81; 95% CI, 1.14–2.89) over 12 years [17], and the other showed that asthmatic adolescents with comorbid attention-deficit hyperactivity disorder (ADHD) but not asthma-alone had an increased risk of developing major depression (HR: 10.25, 95% CI: 3.86–27.19; HR: 2.11, 95% CI: 0.71–6.23, with 7 years of follow-up) [18]. These inconsistent findings might be explained by differences in asthma control, medication use, comorbid conditions, and quality of life across studies. Nevertheless, further studies considering the above factors are needed to better understand the impact of asthma on subsequent elevated depressive symptoms.

Depression may contribute to asthma through a variety of mechanisms. First, depression has been positively associated with high systemic levels of inflammatory mediators (especially IL-4, IL-6 and TNF-a) [16], which have underlying pathogenic roles in asthma. Second, depression has known neuroendocrine effects (i.e. deregulation of the hypothalamic-pituitary-adrenocortical axis and autonomic nervous system), which may exert a link between depression and asthma [19]. Third, depressed individuals tend to be obese and smokers, and these conditions have been demonstrated to independently increase the risk of asthma [20–22]. However, our sensitivity analyses for only including studies adjusted RR with smoking and/or BMI as covariates did not influence the final conclusions, which attenuates the feasibility of this explanation. Fourth, depression has been associated with increased oxidative stress levels and decreased antioxidant functions, and oxidative stress contributes to the pathogenesis of asthma [23,24]. Overall, several mechanisms in patients with genetic susceptibility, either alone or combined, could be implicated in the development of asthma.

This analysis has strengths and limitations. The primary strengths is that this is the first meta-analysis of prospective studies with high quality that explicitly examines the association between depression and asthma based on an exclusive literature search. Studies were excluded if prevalent cases of either depression (for asthma predicting depression onset) or asthma (for depression predicting asthma onset) at baseline were present. This precluded recall bias. We also conducted sensitivity analyses to assess the robustness of our findings. However, the meta-analysis was limited to different adjustments for potential confounders in each study. Although we used fully adjusted estimates from each included study, we cannot exclude the possibility that this factor may affect the final conclusion of the present study. Also, the diagnosis of asthma was based on self-reports without clinical validation among studies for depression predicting incident asthma, which may result in some misclassification bias. However, self-reported asthma has been shown to be valid in epidemiologic studies when questions were asked about physician diagnosis [25]. Furthermore, there are lack of studies from Asian or African ethnicities. Finally, few prospective studies investigate the association between baseline asthma and future risk of depression while excluding prevalent depression. More investigations along these lines are warranted.

In summary, this meta-analysis of prospective studies indicates that depression increases the risk of subsequent adult-onset asthma. However, there is no evidence for a positive association between asthma and incident depression symptoms due to limited data. Further large-scale epidemiologic studies establishing the true association between asthma and subsequent risk of depression, and experimental studies examining the underlying mechanisms linking depression and asthma are warranted.

## Supporting Information

S1 PRISMA Checklist PRISMA Checklist. (DOC)

S1 Table Studies Excluded from the Full-Text Review. (DOCX)
