# Supplementary material for: The Relationship between Depression and Asthma: A Meta-Analysis of Prospective Studies
Source: PLoS One. 2015 Jul 21;10(7):e0132424. doi: 10.1371/journal.pone.0132424 (PMC4510436; doi:10.1371/journal.pone.0132424)
Supplement: S1 Table — (DOCX) [file pone.0132424.s002.docx]

| **Supplemental Table S1. 17 Studies Excluded from the Full-Text Review** | |
| --- | --- |
| **Author** | **Reason for Exclusion** |
| Goodwin RD et al, 2004 [1] | Non-cohort study |
| Jiang CQ et al, 2013 [2] | Non-cohort study |
| Ramos Olazagasti MA et al, 2012 [3] | Non-cohort study |
| Scott KM et al, 2008 [4] | Non-cohort study |
| Trojan TD et al, 2014 [5] | Non-cohort study |
| Wainwright NW et al, 2007 [6] | Non-cohort study |
| Covaciu C et al, 2013 [7] | No relevant outcomes |
| Goodwin RD et al, 2013 [8] | No relevant outcomes |
| Otten R et al, 2009 [9] | No relevant outcomes |
| Slattery MJ et al, 2011 [10] | No relevant outcomes |
| Lucke JC et al, 2010 [11] | No relevant outcomes |
| Katz PP et al, 2010 [12] | No relevant outcomes |
| Hasler G et al, 2006 [13] | Subjects with asthma at study baseline were not excluded for depression predicting asthma |
| Goodwin RD et al, 2009 [14] | Subjects with asthma at study baseline were not excluded for depression predicting asthma |
| Chen MH et al, 2013 [15] | Subjects with depression at study baseline were not excluded for asthma predicting depression |
| Chen MH et al, 2014 [16] | Subjects with depression at study baseline were not excluded for asthma predicting depression |
| Wainwright NW [17] | Depression and risk of asthma admission |

1. Goodwin RD, Fergusson DM, Horwood LJ. Asthma and depressive and anxiety disorders among young persons in the community. *Psychol Med* 2004;34:1465-1474.

2. Jiang CQ, Loerbroks A, Lam KB et al. Mental health and asthma in china: The guangzhou biobank cohort study. *Int J Behav Med* 2013;20:259-264.

3. Ramos Olazagasti MA, Shrout PE, Yoshikawa H, Bird HR et al. The longitudinal relationship between parental reports of asthma and anxiety and depression symptoms among two groups of puerto rican youth. *J Psychosom Res* 2012;73:283-288.

4. Scott KM, Von Korff M, Alonso J et al. Childhood adversity, early-onset depressive/anxiety disorders, and adult-onset asthma. *Psychosom Med* 2008;70:1035-1043.

5. Trojan TD, Khan DA, Defina LF et al. Asthma and depression: The cooper center longitudinal study. *Ann Allergy Asthma Immunol* 2014;112:432-436.

6. Wainwright NWJ, Surtees PG, Wareham NJ et al. Psychosocial factors and asthma in a community sample of older adults. *J Psychosom Res* 2007;62:357-361.

7. Covaciu C, Bergstrom A, Lind T et al. Childhood allergies affect health-related quality of life. *J Asthma* 2013;50:522-528.

8. Goodwin RD, Robinson M, Sly PD et al. Severity and persistence of asthma and mental health: A birth cohort study. *Psychol Med* 2013;43:1313-1322.

9. Otten R, Van de Ven MOM, Engels RCME et al. Depressive mood and smoking onset: A comparison of adolescents with and without asthma. *Psychol Health* 2009;24:287-300.

10. Slattery MJ, Essex MJ. Specificity in the association of anxiety, depression, and atopic disorders in a community sample of adolescents. *J Psychiatr Res* 2011;45:788-795.

11. Lucke JC, Brown W, Tooth L et al. Health across generations: Findings from the australian longitudinal study on women's health. *Biol Res Nurs* 2010;12:162-170.

12. Katz PP, Morris A, Julian L et al. Onset of depressive symptoms among adults with asthma: Results from a longitudinal observational cohort. *Prim Care Respir J* 2010;19:223-230.

13. Hasler G, Gergen PJ, Ajdacic V et al. Asthma and body weight change: A 20-year prospective community study of young adults. *International Journal of Obesity* 2006;30:1111-1118.

14. Goodwin RD, Sourander A, Duarte CS et al. Do mental health problems in childhood predict chronic physical conditions among males in early adulthood? Evidence from a community-based prospective study. *Psychol Med* 2009;39:301-311.

15. Chen M-H, Su T-P, Chen Y-S et al. Higher risk of developing major depression and bipolar disorder in later life among adolescents with asthma: A nationwide prospective study. *J Psychiatr Res* 2014;49:25-30.

16. Chen M-H, Su T-P, Chen Y-S et al. Higher risk of mood disorders among adolescents with adhd and asthma: A nationwide prospective study. *J Affect Disord* 2014;156:232-235.

17. Wainwright NWJ, Surtees PG, Wareham NJ et al. Psychosocial factors and incident asthma hospital admissions in the epic-norfolk cohort study. *Allergy: European Journal of Allergy and Clinical Immunology* 2007;62:554-560.
